# Supplementary material for: Improved Cell Selectivity of Pseudin-2 via Substitution in the Leucine-Zipper Motif: In Vitro and In Vivo Antifungal Activity
Source: Antibiotics (Basel). 2020 Dec 18;9(12):921. doi: 10.3390/antibiotics9120921 (PMC7766124; doi:10.3390/antibiotics9120921)
Supplement: Supplementary file 1 [file antibiotics-09-00921-s001.pdf]

antibiotics

MDPI

Supplementary Materials

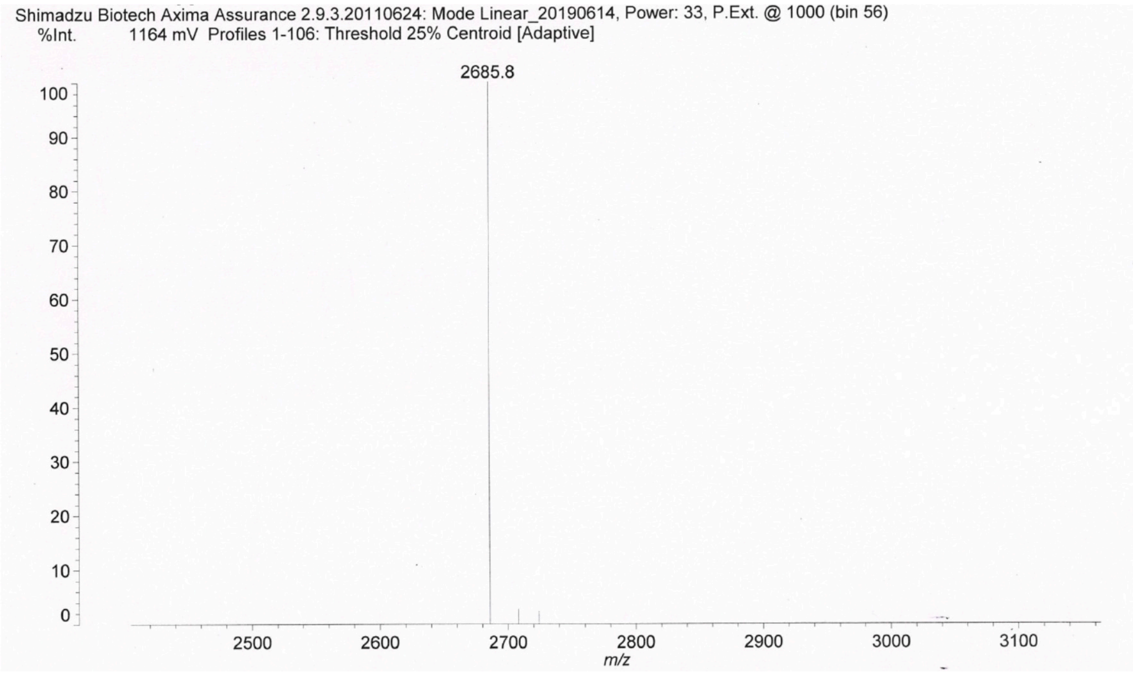

Figure S1. Mass data of pseudin-2.

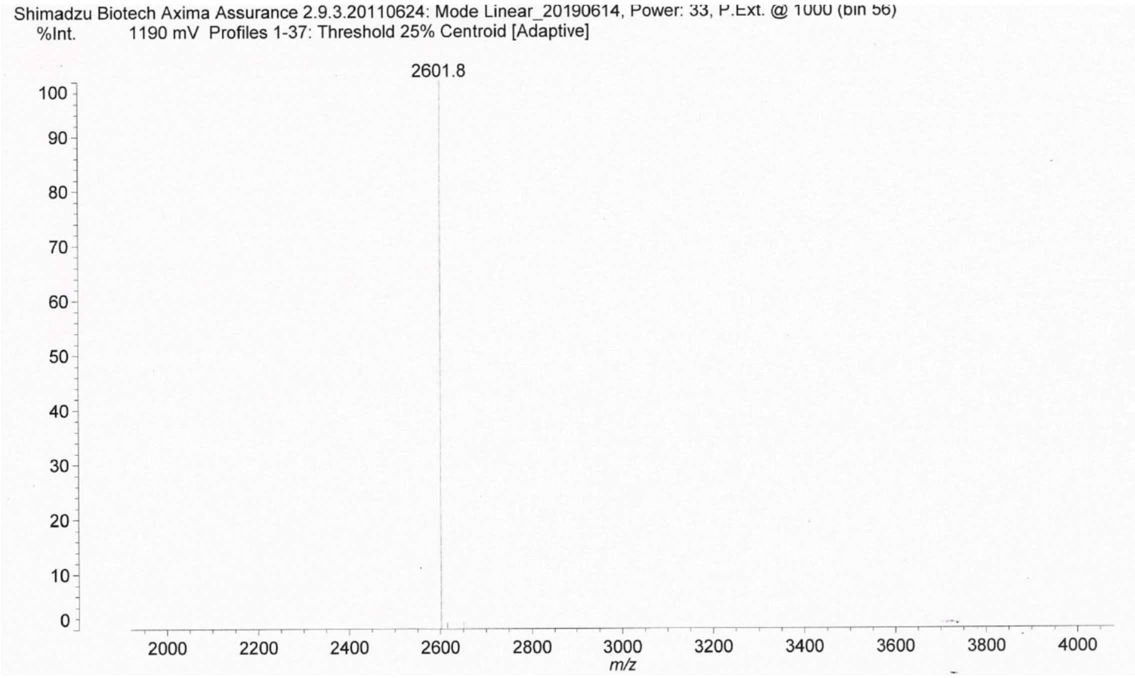

Figure S2. Mass data of P2-LZ1.

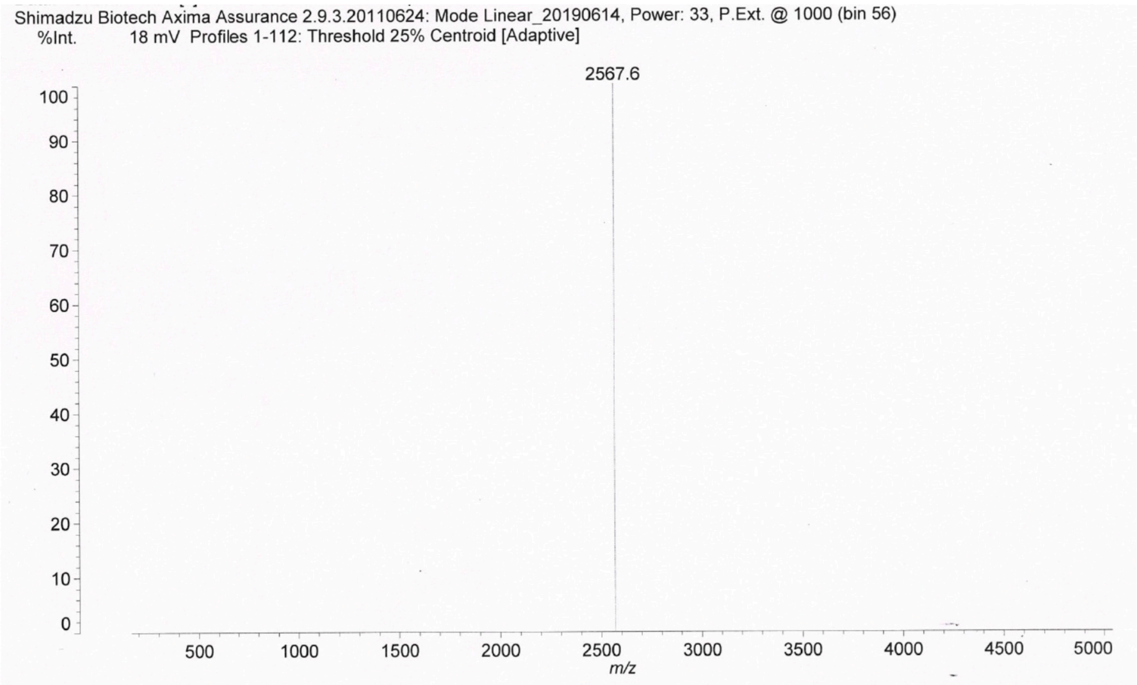

Figure S3. Mass data of P2-LZ2.

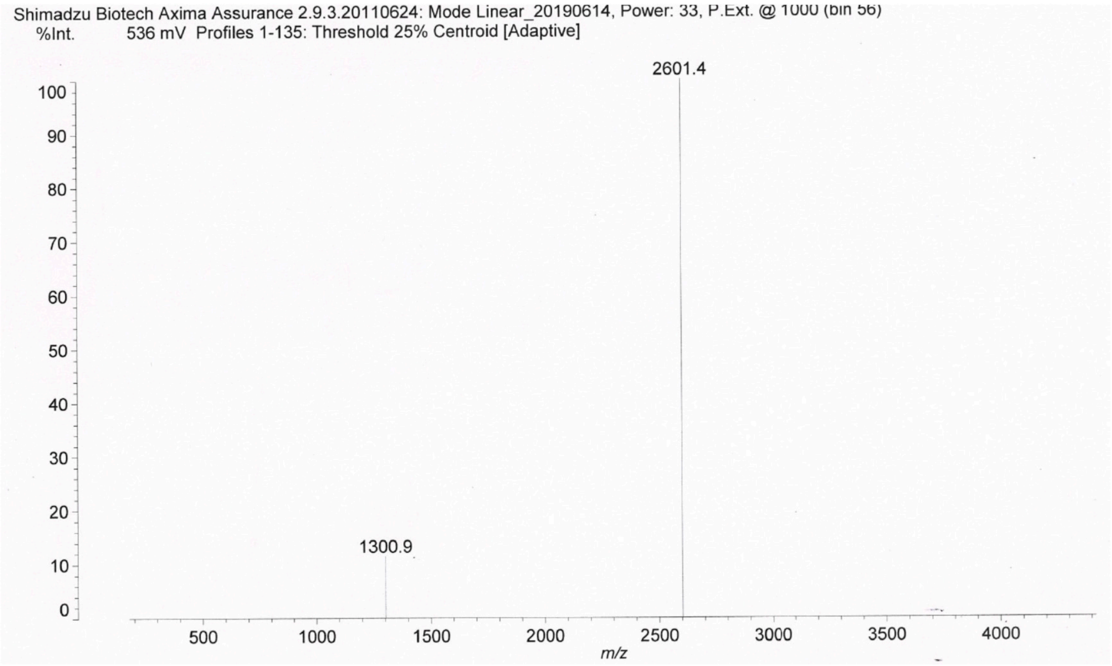

Figure S4. Mass data of P2-LZ3.

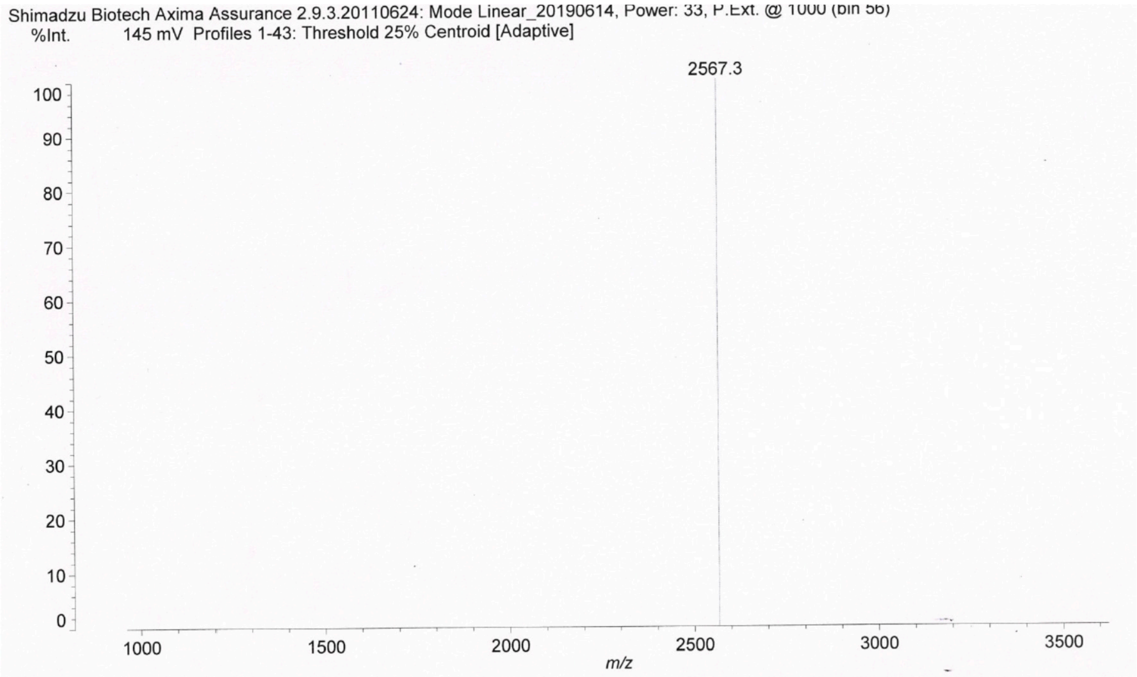

Figure S5. Mass data of P2-LZ4.

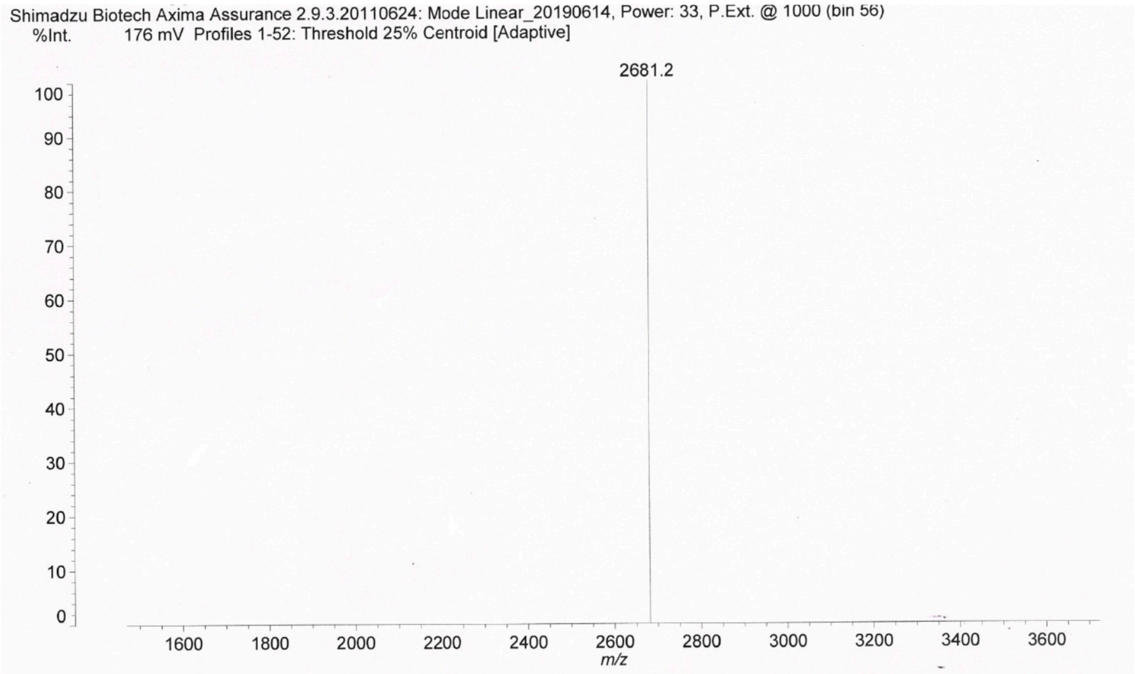

Figure S6. P2-LZ5.

.

HPLC data

==== Shimadzu LCsolution Analysis Report ====

C:\LabSolutions\LCsolution\Sample\B1-2.lcd

Acquired by	: Admin
Sample Name	: Pseudin-2(1)
Sample ID	: 1
Vail #	:
Injection Volume	: 20 uL
Data File Name	: B1-2.lcd
Method File Name	: SCPark-peptide method1.lcm
Batch File Name	:
Report File Name	: Default.lcr
Data Acquired	: 2020-12-01 오후 9:23:05
Data Processed	: 2020-12-01 오후 10:38:08

<Chromatogram>

C:\LabSolutions\LCsolution\Sample\B1-2.lcd

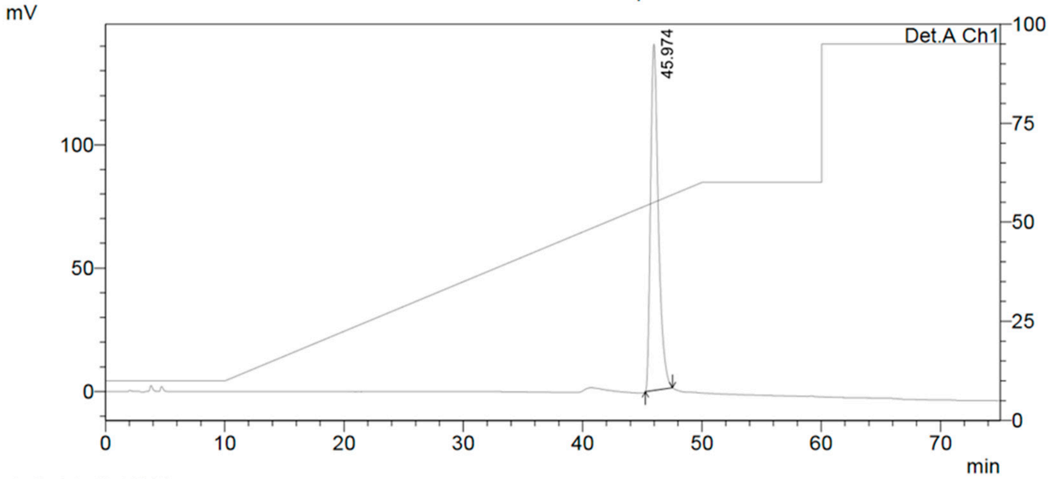

1 Det.A Ch1/215nm

Figure S7. HPLC profile of pseudin-2.

==== Shimadzu LCsolution Analysis Report ====

C:\LabSolutions\LCsolution\Sample\P2-LZ1(1).lcd

Acquired by	: Admin
Sample Name	: P2-LZ1(1)
Sample ID	: 1
Vail #	:
Injection Volume	: 20 uL
Data File Name	: P2-LZ1(1).lcd
Method File Name	: SCPark-peptide method1.lcm
Batch File Name	:
Report File Name	: Default.lcr
Data Acquired	: 2020-11-26 오후 11:26:54
Data Processed	: 2020-11-27 오전 12:41:57

<Chromatogram>

C:\LabSolutions\LCsolution\Sample\P2-LZ1(1).lcd

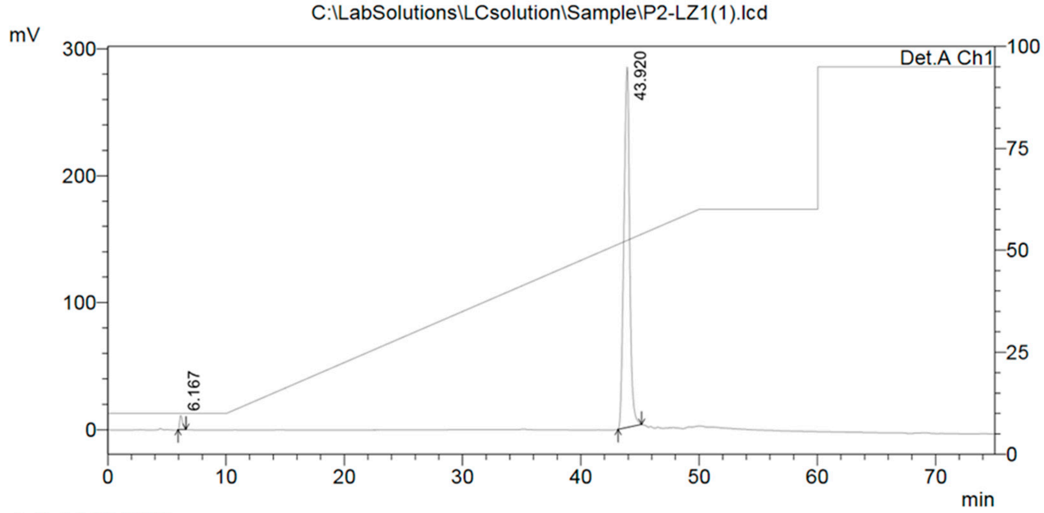

1 Det.A Ch1/215nm

Figure S8. HPLC profile of P2-LZ1.

==== Shimadzu LCsolution Analysis Report ====

C:\LabSolutions\LCsolution\Sample\P2-LZ2(1).lcd

Acquired by	: Admin
Sample Name	: P2-LZ2(1)
Sample ID	: 1
Vail #	:
Injection Volume	: 20 uL
Data File Name	: P2-LZ2(1).lcd
Method File Name	: SCPark-peptide method1.lcm
Batch File Name	:
Report File Name	: Default.lcr
Data Acquired	: 2020-11-27 오전 1:21:01
Data Processed	: 2020-11-27 오전 2:36:03

<Chromatogram>

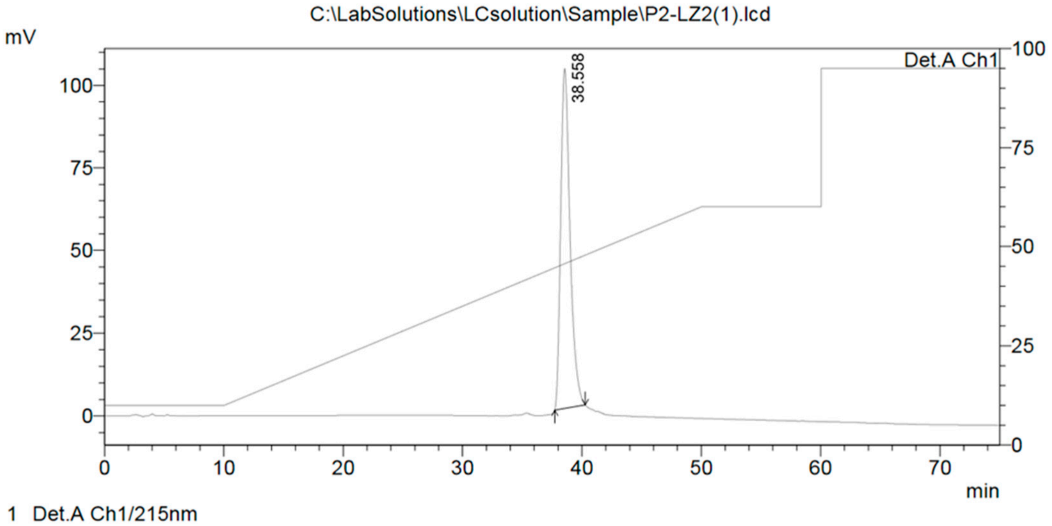

Figure S9. HPLC profile of P2-LZ2

==== Shimadzu LCsolution Analysis Report ====

C:\LabSolutions\LCsolution\Sample\P2-LZ3(1).lcd

Acquired by	: Admin
Sample Name	: P2-LZ3(1)
Sample ID	: 1
Vail #	:
Injection Volume	: 20 uL
Data File Name	: P2-LZ3(1).lcd
Method File Name	: SCPark-peptide method1.lcm
Batch File Name	:
Report File Name	: Default.lcr
Data Acquired	: 2020-11-28 오전 3:31:08
Data Processed	: 2020-11-28 오전 4:46:09

<Chromatogram>

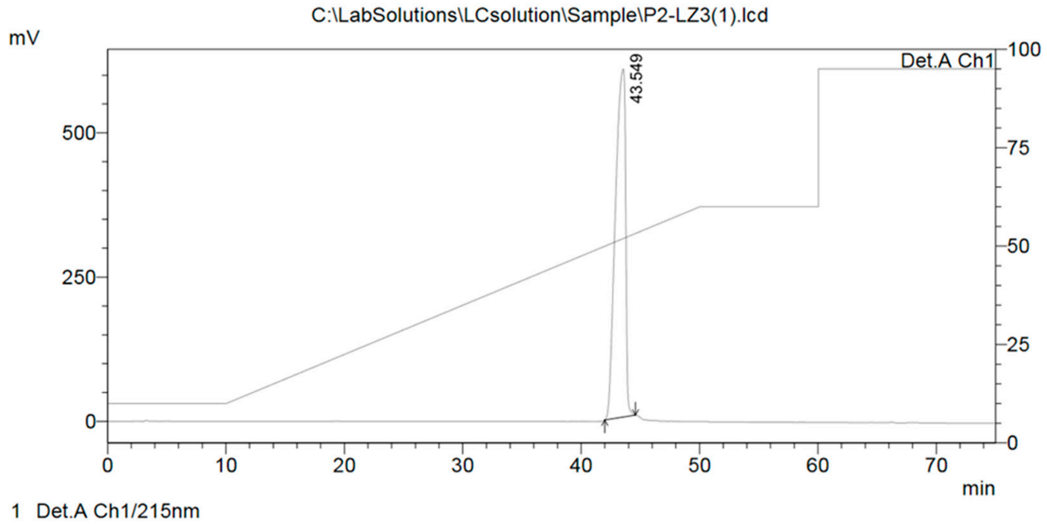

Figure S10. HPLC profile of P2-LZ3

==== Shimadzu LCsolution Analysis Report ====

C:\LabSolutions\LCsolution\Sample\P2-LZ4(1).lcd

Acquired by	: Admin
Sample Name	: P2-LZ4(1)
Sample ID	: 1
Vail #	:
Injection Volume	: 20 uL
Data File Name	: P2-LZ4(1).lcd
Method File Name	: SCPark-peptide method1.lcm
Batch File Name	:
Report File Name	: Default.lcr
Data Acquired	: 2020-11-27 오후 9:53:36
Data Processed	: 2020-11-27 오후 11:08:38

<Chromatogram>

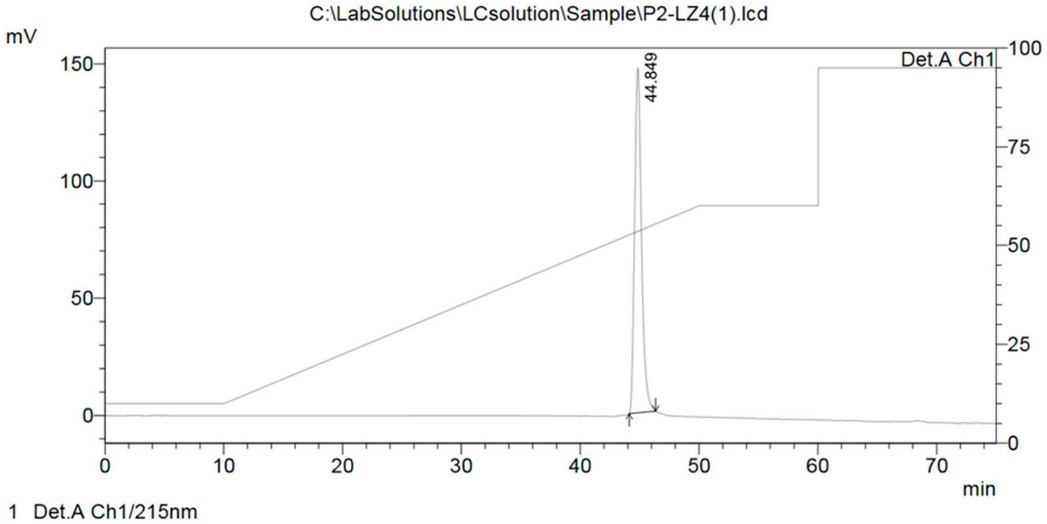

Figure S11. HPLC profile of P2-LZ4

==== Shimadzu LCsolution Analysis Report ====

C:\LabSolutions\LCsolution\Sample\P2-LZ5(1).lcd

Acquired by	: Admin
Sample Name	: P2-LZ5(1)
Sample ID	: 1
Vail #	:
Injection Volume	: 20 uL
Data File Name	: P2-LZ5(1).lcd
Method File Name	: SCPark-peptide method1.lcm
Batch File Name	:
Report File Name	: Default.lcr
Data Acquired	: 2020-11-28 오후 3:58:56
Data Processed	: 2020-11-28 오후 5:13:59

<Chromatogram>

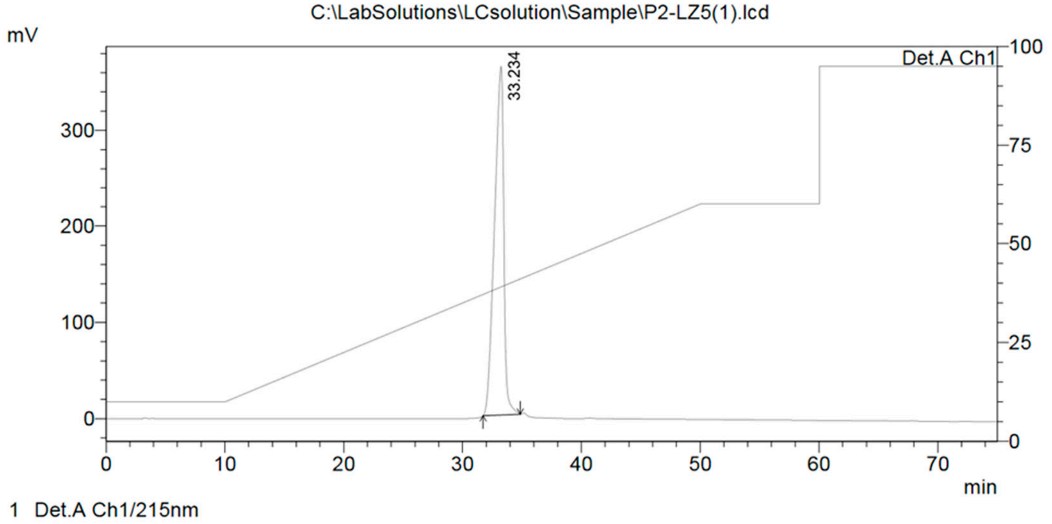

Figure S12. HPLC profile of P2-LZ5

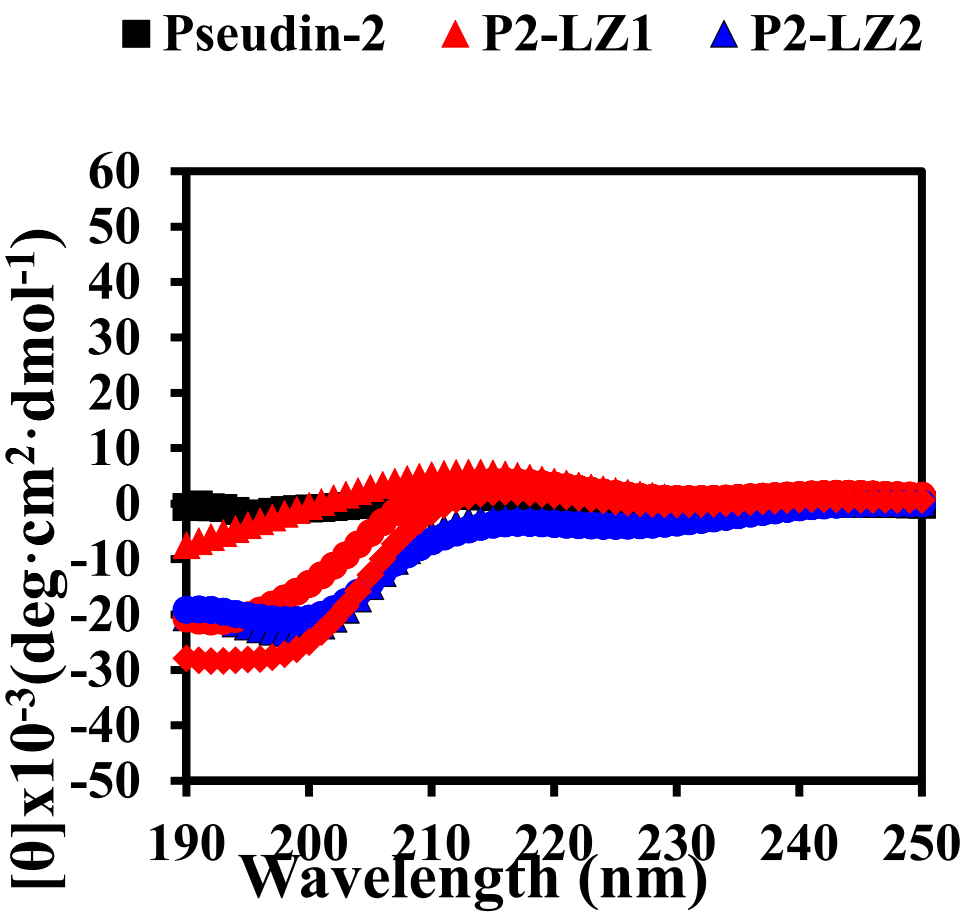

Figure S13. CD spectra of peptides in 10 mM sodium phosphate buffer (pH 7.2).
